# The oxidative phosphorylation inhibitor, atovaquone, upregulates PD-L1 via activation of the ATM/ATR DNA damage response pathway

**DOI:** 10.21203/rs.3.rs-9476762/v1

**Published:** 2026-04-28

**Authors:** Sejal Sharma, Meghana Roy Peddoddi, Anupama Singh, Karla Esbona, Catigan Hedican, Saryn Doucette, Maria Virumbrales-Munoz, Jacques Galipeau, Lisa Barroilhet, Manish Patankar

**Affiliations:** 1 Department of Obstetrics and Gynecology, University of Wisconsin-Madison, Madison, WI, 53705, USA.; 2 William S. Middleton Memorial Veterans Hospital, Madison, WI, 53705, USA; 3 Department of Pathology and Laboratory Medicine, University of Wisconsin-Madison, Madison, WI, 53705, USA; 4 Department of Medicine, University of Wisconsin-Madison, Madison, WI, 53705, USA.; 5 University of Wisconsin Hospitals & Clinics, Madison, WI, 53705, United States.; 6 UW Carbone Cancer Center, University of Wisconsin-Madison, Madison, WI, 53705, USA; 7 Department of Biomedical Engineering, University of Wisconsin-Madison, Madison, WI, 53705, USA

**Keywords:** Atovaquone, PD-L1, Ovarian Cancer, ATM/ATR DNA damage response pathway, Oxidative phosphorylation inhibitor, Combination therapy

## Abstract

Oxidative phosphorylation (OXPHOS), a major metabolic pathway in normal/differentiated cells is also active in tumors and a target for cancer drug development. Atovaquone, an FDA-approved antiprotozoal and OXPHOS inhibitor, blocks electron transport at mitochondrial Complex III resulting in an oxygen radical surge that triggers cancer cell death. Here, we examine mechanisms that attenuate the efficacy of atovaquone as an anti-cancer agent. First, we demonstrate that exposure to atovaquone causes DNA damage and loss of nuclear integrity in cancer cells. DNA damage by atovaquone does not activate cGAS-STING signaling, likely due to repressed cGAS expression in the cell lines tested. Instead, ATM/ATR signaling is activated in response to atovaquone. Recently, we demonstrated that oxidative and endoplasmic reticulum stress in atovaquone-treated cancer cells was associated with elevation in danger associated molecular patterns (DAMPs) corresponding to increased lysis by natural killer cells. Contrary to this immune activating effect, we now report that cancer cells also employ an immunosuppressive mechanism upon exposure to atovaquone. Specifically, we observed ATM/ATR-dependent increase in expression of PD-L1 on the cancer cells. Increase in PD-L1 required STAT1 signaling but was not regulated by IRF1, HIF1α or p53. Increase in PD-L1 was confirmed on peritoneal p53^−/−^ ID8-F3 tumors growing in mice receiving atovaquone therapy. Combining atovaquone with anti-PD-L1 resulted in significant delay in tumor growth. Data from this study provides a mechanistic basis for PD-L1 elevation in tumors treated with atovaquone. Our studies support further development of atovaquone-anti-PD-L1 combination for the treatment of ovarian and other malignancies.

## Introduction

Oxidative phosphorylation (OXPHOS) is a fundamental metabolic pathway responsible for efficient ATP production and maintenance of cellular redox balance in normal cells [1]. Although cancer cells rely on aerobic glycolysis, recent evidence indicates that OXPHOS plays an essential role in tumor growth, survival, and therapeutic resistance [2–4]. In ovarian cancer, mitochondrial respiration promotes metabolic plasticity [5], chemoresistance [6], and immune evasion [7, 8], highlighting OXPHOS as an attractive therapeutic target.

Atovaquone is an FDA-approved anti-malarial drug that inhibits mitochondrial complex III [9] and has emerged as a promising candidate for metabolic targeting in cancer [10]. Previously we have demonstrated that atovaquone exerts potent anti-tumor effects in ovarian cancer model [9, 11]. Studies conducted by other researchers have also shown anti-tumor effects of atovaquone in triple negative breast cancer (TNBC) [12], colorectal cancer [13, 14], gastric cancer [15], non-small cell lung cancer (NSCLC) [16, 17], AML [18], cervical cancer [19], thyroid cancer [20]. Mechanistically, atovaquone disrupts mitochondrial metabolism [19, 20], leading to increased oxidative stress [15, 18] due to excessive accumulation of intracellular reactive oxygen species (ROS) [9, 10, 19, 20], which induce ferroptosis and apoptotic cell death by damaging lipids, proteins and DNA [21–23]. Atovaquone also reduces hypoxia [14, 16] and inhibits pathways associated with tumor aggressiveness [13–15]. Together, these findings indicate that mitochondrial inhibition and ROS-mediated cellular damage within the tumors are key contributors to the anti-cancer activity of atovaquone. Our laboratory recently demonstrated that the oxidative stress induced by atovaquone increases expression of Danger Associated Molecular Patterns (DAMPs), facilitating the recognition of ovarian cancer cells by natural killer (NK) cells [24]. Thus, atovaquone can not only kill the cancer cells by causing high degree of oxidative damage but also contribute to immunologic cell death of the tumors. Previously, we also demonstrated that oxidative stress caused through inhibition of OXPHOS triggers increased expression of the master regulator of anti-oxidative responses, Nrf-2 [25]. More recently we have shown that in response to atovaquone, cancer cells undergo a metabolic shift to the pentose phosphate pathway [25], likely to generate glutathione that can be used to neutralize the intracellular oxygen radicals. Our on-going work is focused on examining these compensatory pathways as they are likely to attenuate the anti-cancer therapeutic efficacy of atovaquone.

From an immunologic standpoint, oxidative stress is a double-edged sword [26]. While it can increase anti-tumor immunity through expression of DAMPs, oxidative stress can also increase expression of immune checkpoints, especially PD-L1 [26–31]. To date, there has not been a systematic study to examine if atovaquone-induced oxidative stress can increase expression of PD-L1 in cancer cells. Here, we identify the molecular mechanisms leading to the expression of this immune checkpoint and provide support for combining atovaquone with anti-PD-L1 antibody as an effective anti-cancer therapeutic strategy.

## Materials and Methods

### Reagents and Cell lines

Unless otherwise specified, all reagents and supplies were from ThermoFisher Scientific (Waltham, MA, USA). Details of antibodies used are listed in Supplementary Table I and all human cell lines were from ATCC (Rockville, MD, USA) and maintained in their recommended media. p53^−/−^ ID8 (ID8 F3) were a gift from Professor Iain McNeish, Imperial College London, UK. All cells were tested quarterly for mycoplasma (Genlantis, Avantor) and profiled for Short Tandem Repeats (STR) every six months.

### Immunocytochemistry / Fluorescence microscopy

Cells cultured in 4-well chamber slides were treated with atovaquone at IC_50_ concentrations (30 μM and 13 μM for OVCAR-5 and ID8-F3, respectively) or vehicle for 24, 48, or 72 h. Cells were then fixed with methanol, permeabilized (0.01% saponin for 10 min at room temperature (RT)), blocked (5% goat serum) and incubated sequentially with unconjugated primary anti-tubulin (overnight at 4 °C) and Alexa Fluor^™^ Plus 555-conjugated goat anti-mouse secondary antibodies (Supplementary Table 1). Cells were then stained for 2 h at RT with PicoGreen (Supplementary Table 2) (3 μg/mL) for 90 min at 37 °C. Cells mounted using ProLong^™^ Gold Antifade Mountant. Fluorescent images were acquired using a Nikon AXR confocal microscope (Tokyo, Japan) equipped with NIS-Elements imaging software. Image analysis and fluorescence quantification were performed using ImageJ (NIH). Four regions of interests (ROI) were selected from each group and nuclear parameters were analyzed using automated particle analysis for perimeter, solidity and aspect ratio (Supplementary Figure 3). Finally, the images in 8-bit format were processed to identify particles and after applying a threshold were converted to binary image.

A standard 2-pixel median filter was applied to remove noise and non-specific signals before using the option “identify particles”. For perimeter and aspect ratio, the size of the particle was adjusted to exclude “salt and pepper” noise or debris. These parameters were analyzed statistically using Two-way ANOVA followed by Dunn’s multiple comparison and Spearmen’s test for heteroscedasticity in GraphPad Prism 10.

### Enzyme Linked Immunosorbent Assay (ELISA)

Levels of 2′3′-cyclicGAMP (2′3′-cGAMP) in conditioned media and cell lysates were quantified using a commercially available 2′3′-cGAMP ELISA kit according to the manufacturer’s instructions. Samples were processed and analyzed in parallel to determine intracellular and extracellular levels of 2′3′-cGAMP.

### Flow Cytometry

For surface staining experiments, cells were incubated with fluorophore conjugated antibodies (Supplementary Table 1) for 30 min at 4 °C in the dark, washed twice with PBS supplemented with 0.02% sodium azide and 0.2% FBS and stained with DAPI (1 μg/mL) for 15 min at RT. For intracellular staining, cells were trypsinized, washed with PBS containing 1% FBS followed by fixation (BD Biosciences, San Jose, CA) for 15 min at 4 °C, washed once with permeabilization/wash buffer (BD Biosciences, San Jose, CA) and incubated with PE conjugated anti-8-OHdG antibody prepared in perm/wash buffer for 1 h at 37°C. Following a final wash, samples were analyzed by using an Attune^™^ NxT Flow Cytometer to quantify median fluorescence intensity (MFI) of 8-OHdG and percentage positive cells. Data acquisition and analysis were performed using FCS Express 7 software.

### Western Blotting

Cells from the various experimental conditions were lysed using RIPA buffer and supplemented with protease and phosphatase inhibitor cocktail. Protein concentrations in the sonicated lysates were determined by microBCA and equal amounts of total protein (10–30 μg per lane) were electrophoresed and transferred to polyvinylidene difluoride (PVDF) membranes. The membranes were blocked using Blocking Buffer and probed with appropriate unconjugated primary and IRDye^®^ conjugated secondary antibodies (Supplementary Table II). Protein bands were visualized using the Odyssey^®^ CLx imaging system and quantified with Image Studio^™^ software (LI-COR Biosciences, Lincoln, NE). Target protein signals were normalized to loading controls (β-actin or α-tubulin) or total proteins and expressed as relative fold change compared with DMSO control.

### Generation of Stable IRF1 Knockdown Cell Lines

OVCAR-5 cells were transduced with three independent IRF1-targeting lentiviral short hairpin RNA (shRNA) constructs that target (5’−3’) GGCTAGAGATGCAGATTAATT, TGGCTAGAGATGCAGATTAAT, CAGAGGTGTACACTAACATTT and a sham control target sequence CCTAAGGTTAAGTCGCCCTCG (VectorBuilder, Chicago, IL) at a multiplicity of infection (MOI) of 7 in the presence of polybrene (10 μg/mL; Vector Builder, Chicago, IL). After 24 h, viral supernatants were replaced with fresh complete growth medium. Cells were then selected with puromycin (2 μg/mL) for 48 h and the surviving cells were harvested, expanded, and cryopreserved for downstream experiments.

### Multiplex Immunohistochemistry (IHC)

Multiplex immunohistochemistry (IHC) was performed using the Ventana Discovery Ultra automated staining platform and Ventana reagents (Ventana Medical Systems, Tucson, AZ). Formalin-fixed, paraffin-embedded (FFPE) tissue sections were deparaffinized followed by heat-induced epitope retrieval (64 min at 95°C) using Cell Conditioner 1. Slides were probed with anti-PD-L1 antibody (1:50) in DaVinci Green Antibody Diluent (BioCare Medical, Pacheco, CA) for 60 min at 37 °C. Following washing with Reaction Buffer, detection was performed using Discovery OmniMap anti-rat horseradish peroxidase (HRP) conjugate for 16 min at 37 °C, with chromogenic visualization achieved using the Discovery ChromoMap DAB Detection Kit. To prevent antibody cross-reactivity prior to subsequent staining, a denaturation step was performed using Discovery Inhibitor. Slides were then incubated with a second primary antibody against EpCAM (Abcam, Waltham, MA) and the incubation and slide visualization was conducted under conditions identical to those described for anti-PD-L1 antibody.

Multiplexed images were acquired using the Nuance Multispectral Imaging System (PerkinElmer, Waltham, MA) at 40X. A spectral library was used to unmix the multispectral images allowing the separation of PD-L1, EpCAM and hematoxylin signals. Five region of interests per tumor section were manually selected by excluding necrotic areas, stromal compartments and tissue artifacts. Using inform software (version 2.4.8), PD-L1 positivity (%) and mean staining intensity were quantified within EpCAM positive and or total selected ROI. Identical threshold settings were applied across all groups to ensure unbiased comparison.

### Animal Studies

All animal experiments were conducted in accordance with protocols approved by the Institutional Animal Care and Use Committee (IACUC) of the University of Wisconsin-Madison (Protocol number: M006730). Female C57BL/6 mice were housed in the University’s Small Animal Facility under specific pathogen-free conditions with ad libitum access to food and water.

Luciferase expressing ID8-F3 tumors (1 × 10^6^) were injected intraperitoneally (I.P.) in female 6–8 weeks old C57BL/6 (n = 5 per group) and tumor establishment was monitored by chemiluminescence imaging (LagoX, Spectral Instruments Imaging, Tucson, AZ). To monitor PD-L1 expression in response to atovaquone, mice were treated with either atovaquone (200 mg/kg) or vehicle control (castor oil) by oral gavage five times per week for two weeks. At the experimental endpoint, mice were euthanized, and tumor tissues were harvested, fixed in 10% neutral buffered formalin for 24 h, and subsequently transferred to 70% ethanol. Fixed tissues were processed and analyzed for PD-L1 and EpCAM expression by multiplex IHC.

To evaluate the therapeutic efficacy of combining atovaquone with PD-L1 blockade, female C57BL/6 mice were randomly assigned to four treatment groups (n = 5/4 per group): control (castor oil + rat IgG), atovaquone alone (200 mg/kg), anti PD-L1 alone (2.5 mg/kg), and combination therapy (atovaquone 200 mg/kg + anti PD-L1 2.5 mg/kg). Atovaquone was administered by oral gavage for five days/week for five weeks. Anti-PD-L1 or its isotype control were administered I.P. beginning at week 3 after start of atovaquone dosing, with five doses injected every four days (200 mg/kg). Animals reaching predefined endpoints, including signs of pain and distress like hunched posture, tumors impeding mobility, respiratory distress were humanely euthanized in compliance with approved animal care protocol.

### Statistical Analysis

Statistical analyses were performed using GraphPad Prism version 10. Data presented as mean ± standard deviation (SD) from at least three independent experiments unless otherwise indicated. Comparisons between two groups were performed using an unpaired Student’s *t*-test. For experiments involving multiple groups, one-way or two-way analysis of variance (ANOVA) was used, followed by appropriate post-hoc tests. A p value < 0.05 was considered statistically significant.

## Results

### Atovaquone causes oxidative stress-induced DNA damage in ovarian cancer cells

**Figure 1:**
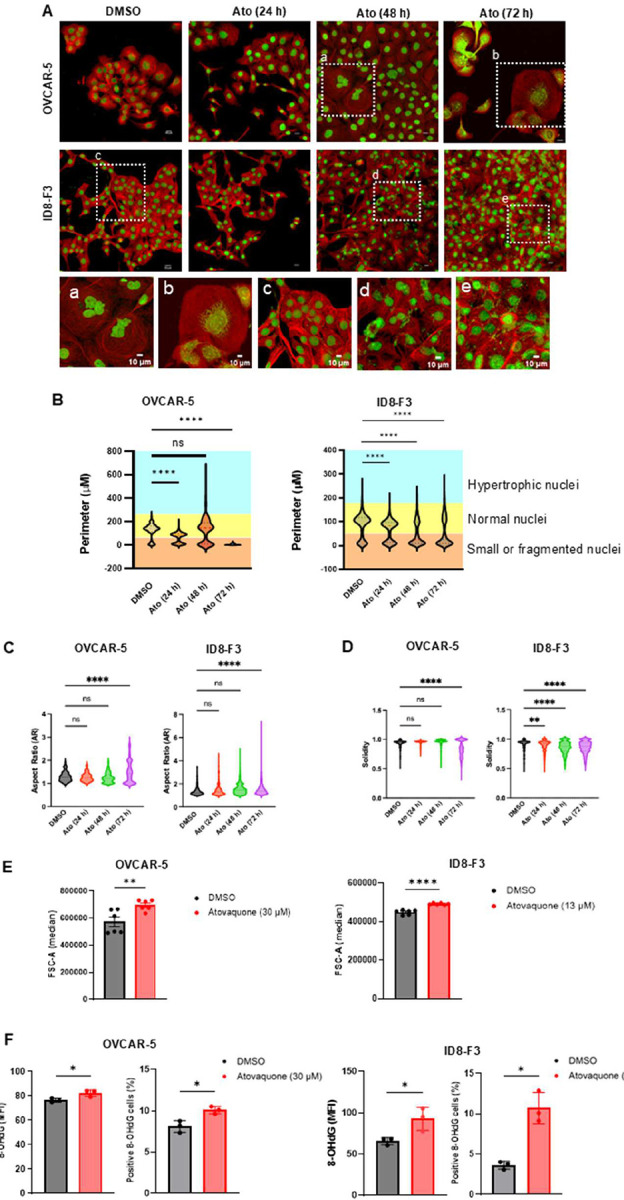
Atovaquone induced oxidative stress causes nuclear DNA damage: *A*, Temporal assessment of DNA fragmentation, nuclear morphology and micronuclei formation in atovaquone (Ato)-treated OVCAR-5 (at IC_50_ = 30 μM) and ID8-F3 cells (at IC_50_ = 13 μM). Cells stained with anti-α-tubulin (red) and the DNA binding dye, picogreen (green) for 24, 48, and 72 h were monitored by fluorescent microscopy (Scale bar – 50 μm). *a-e*, Digitally magnified inset regions from corresponding original images, to show nuclear alterations in the atovaquone treated cells compared to controls (Scale bar – 10 μm). *B*, Quantitative assessment of nuclear perimeter distinguishes normal (yellow), small or fragmented (orange) and hypertrophic nuclei (blue) in vehicle control and atovaquone-treated cells. *C and D*, show quantitative data on aspect ratio and solidity of nuclei of vehicle control and atovaquone-treated cells, respectively. *E*, Flow cytometry data quantifying median forward scatter (FSC-A) as a measurement of cell size after 72 h with atovaquone *F*, Data from flow cytometry experiment assessing mean fluorescence intensity (MFI) and percentage of cells positive for 8-hydroxy-2’-deoxyguanosine (8-OHdG) after 72 h treatment with atovaquone compared to corresponding vehicle control. Two-way ANOVA with Dunnet’s multiple comparison *(B-D)* or student T tests *(E-F)* were performed to compare mean ± standard deviation. For *A-F*, N=3 biological replicates (with each replicate containing three technical replicates) for each experimental condition. Statistical significance was defined as p < 0.05 (ns: p > 0.05, *p ≥ 0.05, **p ≥ 0.01, ***p ≥ 0.001, ****p ≥ 0.0001)

Picogreen staining of atovaquone treated OVCAR-5 and ID8 F3 cells showed noticeable structural alterations within the nucleus and chromatin region including enlarged nuclei, non-homogenous distribution of chromatin and nuclear fragmentation (Fig.1A). We quantified these phenotypic changes using three shape descriptors, nuclear perimeter, aspect ratio and solidity.

Both cell lines tested showed responses in three phases (Fig. 1B). At 24 h, both cell lines showed significant decrease in perimeter due to small compact nuclei suggesting early stress. By 48 h, OVCAR-5 and ID8 F3 showed nuclear hypertrophy and nuclear aggregation with OVCAR-5 displaying high variability with “long tails” reaching 700 μm. By 72 h, perimeters for both cell lines dropped towards baseline, indicating significant cell death/fragmentation.

At 72 h, both cell lines showed a significant increase in the nuclear aspect ratio indicating changes in the shape of the nuclei (Fig. 1C). These results demonstrate a transition from a well-rounded nucleus to an oblong shape, a hallmark of physiological stress. At 72 h, both cell lines also showed significant decrease in solidity from 1.0 towards 0.5 suggesting non-homogeneous chromatin and nuclear membrane irregularities which is consistent with an apoptotic nuclear phenotype (Fig. 1D). Altogether, our data suggests that cancer cells after treatment with atovaquone show nuclear hypertrophy, nuclear blebbing and chromatin reorganization indicating apoptosis.

These changes in nuclear morphology were associated with alterations in cell size. We measured forward scatter area (FSC-A) as a parameter for cell swelling by flow cytometry. Quantitative analysis revealed a significant increase in median FSC-A in atovaquone treated OVCAR-5 and ID8 F3 cells compared to DMSO control, indicative of cell swelling. (Fig. 1E).

To directly evaluate oxidative DNA damage, levels of 8-hydroxy-2′-deoxyguanosine (8-OHdG), a well-established marker of oxidized DNA, were quantified by flow cytometry. A significant increase in 8-OHdG MFI and the percentage of 8-OHdG positive cells were observed in cells treated with atovaquone compared to DMSO control (Fig. 1F). These results demonstrate that atovaquone causes oxidative DNA damage.

### Atovaquone upregulates cell surface PD-L1 expression in ovarian cancer cells

**Figure 2:**
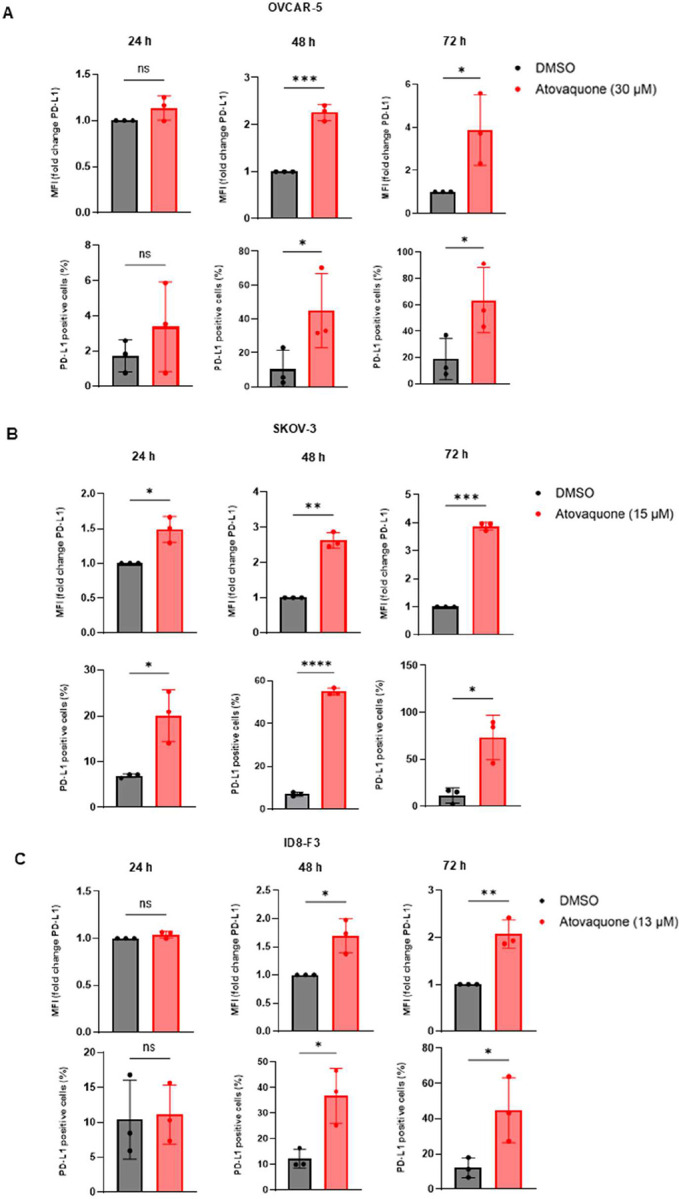
PD-L1 expression increases after atovaquone treatment: *A-C*, Bar graphs show flow cytometry assessment of Mean Fluorescence Intensity (MFI) and percent PD-L1 positivity in atovaquone-treated versus vehicle controls at three timepoints for OVCAR-5, SKOV-3 and ID8-F3 cells, respectively. For comparison, unpaired T-test were performed with mean ± standard deviation for each measurement. For *A-C*, N=3 biological replicates (each replicate containing three technical replicates) for each experimental condition. Statistical significance was defined as p < 0.05 (ns: p > 0.05, *p ≥ 0.05, **p ≥ 0.01, ***p ≥ 0.001, ****p ≥ 0.0001).

Atovaquone treatment of two human cancer cell lines, OVCAR-5 and SKOV3 showed a time dependent increase in PD-L1 expression (Fig. 2A–B). Similar results were observed in ID8-F3 murine ovarian cancer cells suggesting a conserved response across multiple cancer cell lines (Fig. 2C).

**Figure 3:**
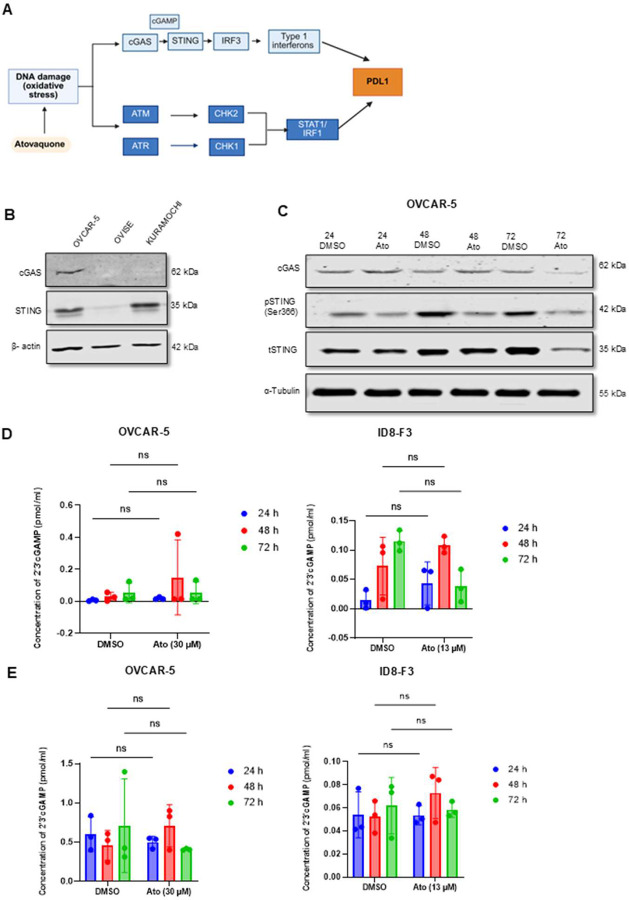
cGAS-STING was not up-regulated in atovaquone treated ovarian cancer cells: *A*, cGAS-STING and ATM/ATR signaling pathways (created with BioRender.com) triggered by oxidative stress that led to increased expression of PD-L1. *B*, Representative western blots showing endogenous cGAS and STING expression in OVCAR-5, OVISE and Kuramochi human ovarian cancer cells. *C*, Representative western blots of cGAS, total and phosphorylated STING and α-tubulin (loading control) in OVCAR-5 cells treated with atovaquone (Ato) for 24, 48 and 72 h. *D and E*, Amount of 2’,3’-cGAMP conditioned media or in cell lysates of OVCAR-5 and ID8-F3 cells treated at three timepoints with atovaquone (Ato) compared to vehicle controls, respectively. For *D and E*, two-way ANOVA with Sidak’s multiple comparison was performed on the mean ± standard deviation for the 2’3’cGAMP data. For *B-E*, N=1 biological replicates for each experiment. Statistical significance was defined as p < 0.05 (ns: p > 0.05, *p ≥ 0.05, **p ≥ 0.01, ***p ≥ 0.001, ****p ≥ 0.0001)

To delineate the DNA damage response (DDR) mechanisms activated by atovaquone, we examined cGAS-STING and ATM/ATR pathways that are known to upregulate PD-L1 expression in stressed cells (Fig. 3A) [32] [33]. First, we assessed basal cGAS and STING protein expressions in a selected panel of human cancer cells by western blot. OVCAR-5 cells expressed both cGAS and STING proteins, whereas Kuramochi and OVISE cells showed cGAS and/or STING deficiency (Fig. 3B). Given the presence of both pathway components, OVCAR-5 cells were selected for further analysis. In OVCAR-5 cells, cGAS and phospho-STING (pSTING) levels were downregulated after atovaquone treatment (Fig. 3C). Furthermore, the second messenger cGAMP (Fig. 3D–E) and IFNβ were undetected (data not shown) in the media or cell lysates in atovaquone treated OVCAR-5 and ID8-F3 cells. Altogether these findings suggest that atovaquone induced DNA damage does not activate cGAS-STING pathway in the cancer cells tested in this study.

### Atovaquone activation of ATM/ATR signaling pathway is required for expression of PD-L1

**Figure 4:**
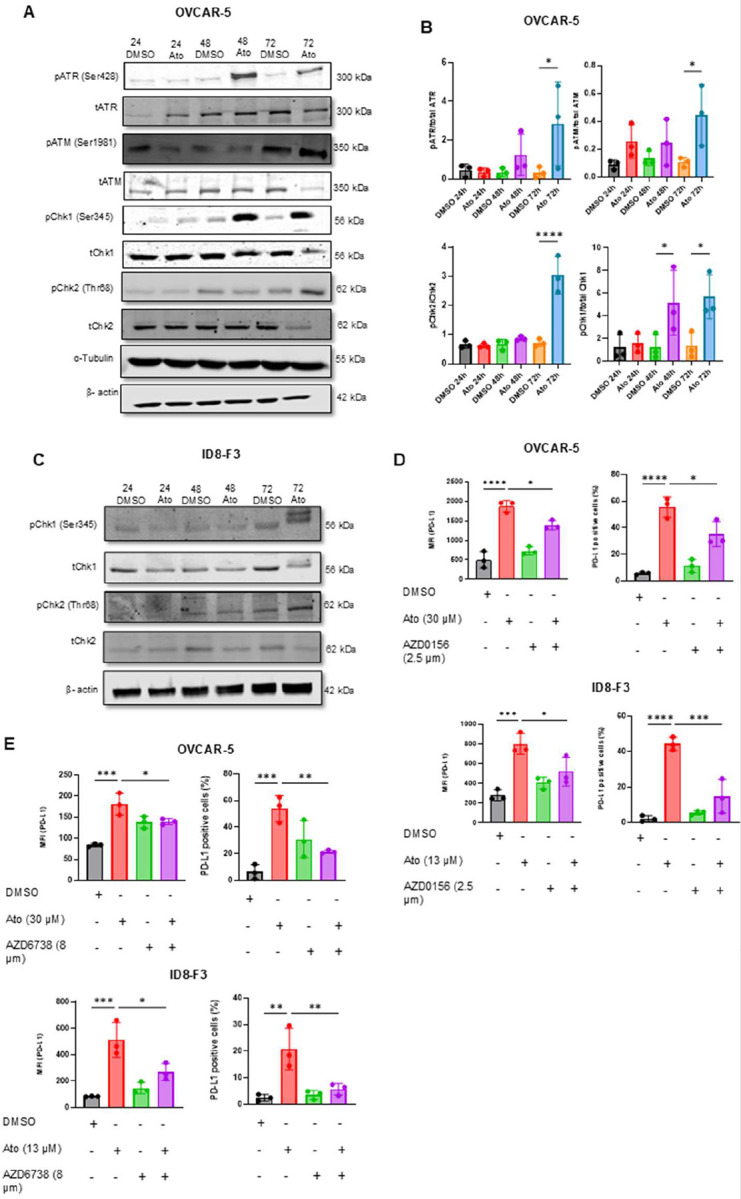
ATM/ATR required for PD-L1 expression in atovaquone treated ovarian cancer cells: *A*, Representative western blot show changes in the expression of phosphorylated ATM, ATR, Chk1 and Chk2 (pATM, pATR, pChk1 and pChk2, respectively) compared to their corresponding total proteins (tATM, tATR, tCHK1 and tCHK2) in OVCAR-5 cells treated with 30 μM atovaquone (Ato) for three timepoints are shown. *B*, Protein levels of p-ATM, p-ATR, pChk1 and pChk2 and its total proteins were quantified by densitometry and normalized by total protein or housekeeping genes to measure fold change. *C*, Representative western blots show changes in expression of pChk1 and pChk2 compared to the corresponding total proteins in ID8-F3 cells treated with 13 μM atovaquone (Ato). *D and E*, Flow cytometry data measuring Mean Fluorescence Intensity (MFI) and percent PD-L1 positivity in OVCAR-5 and ID8-F3 cells pretreated with ATM (AZD0156) and ATR (AZD6738) inhibitors, respectively, followed by atovaquone (Ato) are shown. cell For *B, D and E*, one-way ANOVA with Sidak’s multiple comparison was used to compare mean ± standard deviation. N=3 biological replicates for each experiments condition. Statistical significance was defined as p < 0.05 (ns: p > 0.05, *p ≥ 0.05, **p ≥ 0.01, ***p ≥ 0.001, ****p ≥ 0.0001)

Immunoblot analysis confirmed that atovaquone induced oxidative stress activates ATM/ATR signaling pathway. In OVCAR-5, we observed an increase in phosphorylation of ATM (Ser1981), ATR (Ser428), and their respective downstream kinases Chk2 (Thr68) and Chk1 (Ser345) (Fig. 4A–B). Similar activation of Chk1 and Chk2 was observed in murine ID8-F3 cells (Fig. 4C). To confirm if ATM/ATR regulates PD-L1 expression, OVCAR-5 and ID8-F3 cells were treated with atovaquone alongside selective ATM (AZD0156) or ATR (AZD6738) inhibitors. Flow cytometry analysis revealed that ATM inhibition reduced surface PD-L1 by 22.8% in OVCAR-5 and 44.3% in ID8-F3 cells (Fig. 4D). ATR inhibition also resulted in robust decrease in PD-L1 expression by 46.6**%** and 69.9%, respectively (Fig. 4E). Collectively, these data demonstrate that atovaquone-induced PD-L1 upregulation is driven by the ATM/ATR signaling pathway.

### STAT1 is a major transcriptional regulator of PD-L1 expression following atovaquone treatment

**Figure 5:**
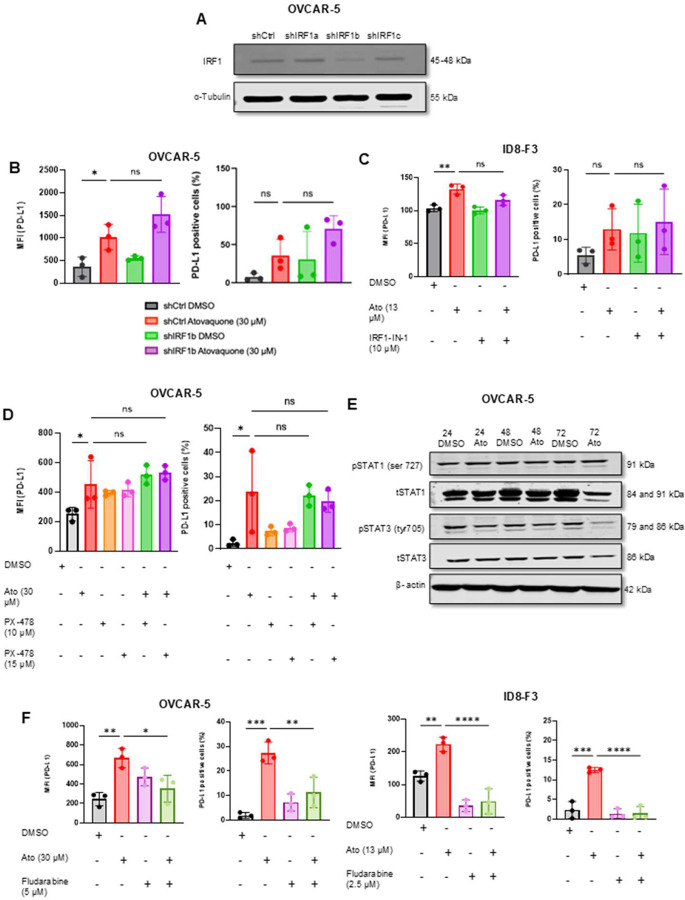
STAT1 is an important transcription factor in atovaquone treated ovarian cancer cells: *A*, Representative western blot of total IRF1 expression in OVCAR-5 transduced with control (shCtrl) and three different IRF1 shRNA (shIRF1a, shIRF1b and shIRF1c) is shown. α-Tubulin used as loading control. *B*, Flow cytometry data showing Mean Fluorescence Intensity (MFI) and percent positivity for PD-L1 in control and IRF-1 knockdown cells after treatment with atovaquone for 72 h are presented. *C*, ID8-F3 cells pretreated with IRF1 inhibitor (IRF-IN-1) followed by 48 h exposure to atovaquone (Ato) were monitored for PD-L1 expression by flow cytometry to measure MFA and percent positivity. *D*, OVCAR-5 parental cells were pretreated with HIF1α inhibitor (PX-478) at two concentrations for 48 h and PD-L1 expression determined by flow cytometry. *E*, OVCAR-5 cells treated with atovaquone for 24, 48 and 72 h. Representative western blot of p-STAT1, p-STAT3, and their corresponding total proteins are shown. (F) Parental OVCAR-5 and ID8-F3 cells were pretreated with STAT1 inhibitor, fludarabine following 48 h atovaquone treatment and PD-L1 expression was monitored by flow cytometry. One-way ANOVA with Sidak’s multiple comparison was performed on the means ± standard deviation. For *A-F*, N=3 biological replicates for each experimental condition. Statistical significance was defined as p < 0.05 (ns: p > 0.05, *p ≥ 0.05, **p ≥ 0.01, ***p ≥ 0.001, ****p ≥ 0.0001)

To identify the transcriptional regulators responsible for PD-L1 induction, we systemically investigated several factors (Fig. 5). We first investigated IRF1 which is a canonical regulator of PD-L1 expression [34]. Neither shRNA mediated IRF1 knockdown in OVCAR-5 (shIRF1b) (Fig. 5A, Supplementary Fig. 1A, B) nor pharmacological inhibition (IRF1-IN-1) in ID8-F3 cells reversed the atovaquone-mediated increase in PD-L1 expression (Fig. 5B–C). These results suggest that IRF1 is not a primary mediator of PD-L1 expression. Additionally, p53 deficient cell lines including ID8-F3 and SKOV3 showed robust PD-L1 induction suggesting p53 involvement in increasing the expression of this immune checkpoint following atovaquone treatment was also unlikely (Fig. 2B, C). Furthermore, PD-L1 expression was not affected after HIF1α inhibition in OVCAR-5 and shIRF1b OVCAR-5 cells (Fig. 5D, Supplementary Fig.1C).

Consistent with other studies [35, 36], we observed reduced pSTAT3 after atovaquone treatment (Fig. 5E) suggesting that STAT3 activation is unlikely to mediate PD-L1 induction. We did observe an increase in pSTAT1 following atovaquone treatment (Fig. 5E), implicating STAT1 as a potential contributor. To confirm, OVCAR-5 and ID8-F3 cells were treated with the STAT-1 inhibitor, fludarabine, following atovaquone treatment. Inhibition of STAT-1 resulted in near complete abrogation of PD-L1 expression (Fig. 5F). Given that STAT1 typically functions upstream of IRF1, we simultaneously inhibited STAT1 in parental OVCAR-5 and OVCAR-5 (siIRF1b) cells and did not see further decrease in PD-L1 levels. Simultaneous inhibition of STAT1 and IRF1 by inhibitors in ID8 F3 cells showed reduced PD-L1 expression (Supplementary Fig. 1D, E). Collectively, these findings demonstrate that STAT1 is the dominant regulator of atovaquone-induced PD-L1.

### Atovaquone in combination with anti PD-L1 therapy reduces tumor growth in a syngeneic ovarian cancer mouse model

**Figure 6:**
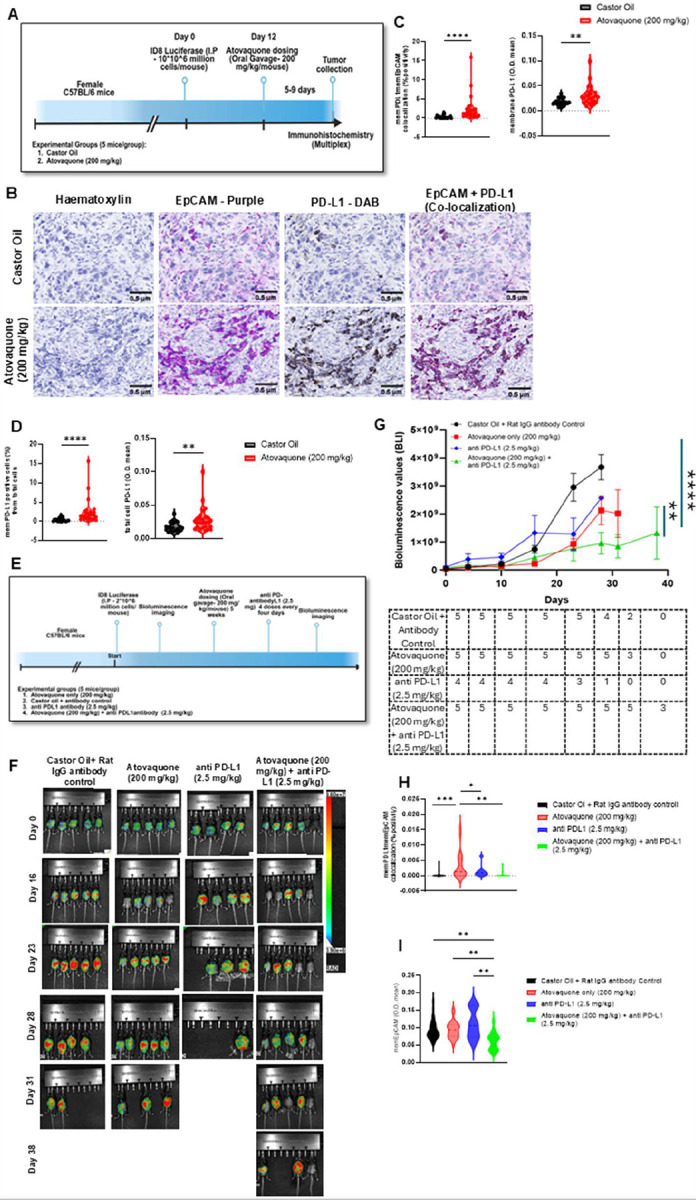
Atovaquone enhances the efficacy of anti-PD-L1 treatment in ID8-F3 ovarian cancer model: *A*, Schematic representation of the experimental design and treatment schedule for in vivo study of PD-L1 expression in atovaquone treated ID-F3 tumors. *B*, Representative IHC images of PD-L1 expression in tumors collected from control and atovaquone treatment groups. IHC was conducted on paraffin embedded tumors by staining with anti-PD-L1 and anti-EpCAM antibodies. Hematoxylin was used as nuclear stain and images were collected at 40X. *C and D*, Five regions of interest of the tumors were selected and analyzed using inForm software to quantify EPCAM and PD-L1 expression. Mann Whitney tests were performed with mean ± standard deviation. Statistical significance was defined as p < 0.05 (ns: p > 0.05, *p ≥ 0.05, **p ≥ 0.01, ***p ≥ 0.001, ****p ≥ 0.0001). *E*, Schematic representation of the experimental design and treatment schedule for testing combination of atovaquone with anti PD-L1 therapy. *F and G*, Tumor growth was quantitatively analyzed at the designated timepoints by bioluminescence imaging of the peritoneally implanted Luc-ID8-F3 tumors. Total emission (photons/sec) was calculated by Aura software and values at each time point were normalized to the emission measurements for the same mouse prior to initiating treatment. Statistical significance was determined by two-way ANOVA followed by Tukey’s multiple comparison test. Data were indicated as the mean ± standard error from mean. *H and I*, quantification of PD-L1 and EpCAM colocalization *(H)* and EpCAM only *(I)* in tumors from the different treatment cohorts determined from IHC staining. One-way ANOVA with multiple comparison tests was performed. Statistical significance was defined as p < 0.05 (ns: p > 0.05, *p ≥ 0.05, **p ≥ 0.01, ***p ≥ 0.001, ****p ≥ 0.0001)

To validate our in vitro findings of PD-L1 upregulation, we assessed the effect of atovaquone on ID8-F3 syngeneic mouse ovarian cancer model. Following two weeks of treatment, IHC analysis revealed a significant increase in both percentage of PD-L1 positive cells and the intensity of PD-L1 expression compared to controls (Fig. 6A–C) Furthermore, increased co-localization of EpCAM and PD-L1 was also observed in atovaquone treated group, indicating that PD-L1 upregulation occurred within tumor epithelial compartment (Fig. 6C).

Next, we assessed whether atovaquone-induced PD-L1 expression influenced responsiveness to immune checkpoint blockade. Mice were treated according to the regimen outlined in Fig. 6E. While PD-L1 monotherapy showed limited efficacy with mice reaching high tumor burden rapidly, the combination of atovaquone with anti-PD-L1 significantly reduced tumor growth compared to vehicle or atovaquone alone (Fig. 6F ,G, I). While there was a progressive increase in tumor burden in the combination group, three of the five animals were alive at day 38 of the experiment. It is important to note that one of the three mice surviving at day 38 in the combination group showed low tumor burden from start (Day 0) of the experiment and hence may represent an outlier due to lack of sufficient engraftment of the ID8-F3 tumor cells. Overall, however, bioluminescence measurements showed lower levels of tumor burden at this day 38 timepoint in the combination group as compared to the atovaquone-only treated group.

Positive effects of the atovaquone plus anti-PD-L1 combination therapy were also indicated in the immunohistology analysis of tumors excised from 3–5 mice from the control and the treatment groups. Tumors from the atovaquone-only group showed an increase in PD-L1 expression on the EpCAM-expressing cells (Fig. 6H). This increase was statistically significant as compared to the control, providing further evidence of an increase in PD-L1 expression in vivo in response to atovaquone. Mice receiving atovaquone plus anti-PD-L1 showed very low level of PD-L1 on their EpCAM expressing cells (Fig. 6H). This decrease corresponds to an overall decrease in EpCAM positive ID8-F3 cells in the combination treatment group suggesting enhanced elimination of the tumors in this group as compared to the atovaquone-only cohort (Fig. 6I).

In the anti-PD-L1 group, we observed surprising level of toxicity with two mice succumbing to high tumor burden and the remaining two animals dying within 30 minutes of receiving the fourth dose of anti-PD-L1 antibody (Fig. 6G). Interestingly, when combined with atovaquone, the toxicity of anti-PD-L1 appeared to be significantly attenuated. Since our study was not designed to address this issue, the potential protective effect of atovaquone against anti-PD-L1 toxicity needs further examination. To assess treatment-related toxicity, liver tissues were examined histologically. Nodular regenerative hyperplasia, a lesion associated with portal venopathy, systemic disease, and drug toxicity, was present in both control and treated groups, indicating it is unlikely to be treatment related. Mild lymphocytic hepatic infiltrates were more frequent in PD-L1–treated mice than in controls, consistent with a potential immune-mediated treatment effect. No hepatocyte necrosis, apoptosis, or interface hepatitis was observed. In the absence of serum transaminase measurements, the clinical significance of the inflammation remains unclear. Treatment with atovaquone or combination group alone was not associated with histologic abnormalities (Table 1).

Collectively, these in vivo data support the functional relevance of atovaquone induced PD-L1 upregulation and demonstrate that atovaquone in combination with anti PD-L1 antibody is an effective approach compared to individual treatment in ovarian cancer model, ID8-F3, used in our studies.

## Discussion

In this study, we provide mechanistic understanding of a major immune suppression strategy that may limit the efficacy of atovaquone as an anti-cancer agent. By inhibiting electron transport in the mitochondria, atovaquone induces an intracellular surge in oxygen radicals [9, 37]. With cancer cells experiencing chronic oxidative stress [38], further increase in oxygen radicals by atovaquone results in cellular pathology that is difficult for the cancer cells to overcome using their already stressed antioxidant mechanisms. The lower chronic oxidative stress in healthy cells allows them to sustain the oxygen radical flux generated by atovaquone. Atovaquone, being an FDA-approved agent, is therefore a strong candidate to exploit this vulnerability of the cancer cells. Several translational and clinical studies are currently underway to repurpose atovaquone as an anti-cancer agent [39–42]. Our recent study has further shown that the oxidative damage caused by atovaquone in ovarian cancer cells results in an increase in the expression of the DAMPs, HMGB1, TFAM and calreticulin [24]. We have further demonstrated that the activating natural killer cell receptors, NKG2D and NKp46 recognize atovaquone-treated cancer cells and is likely the reason for their increased killing by human natural killer cells. These observations suggest that atovaquone can cause cancer cell death by oxidative damage and increased immune recognition of the damaged cancer cells.

Our studies confirm that atovaquone upregulates PD-L1 in cancer cells. This observation compliments previous studies where combined use of atovaquone and anti-PD-L1 increased survival of mice implanted with CT26 and 4T1 tumors [43]. Our studies, for the first time demonstrate that atovaquone increases the expression of PD-L1 in cancer cells, providing direct evidence and support for combining this agent with anti-PD-L1 or anti-PD-1 therapy.

Emerging studies have attributed the positive benefits of atovaquone and anti-PD-L1 combination therapy to reduction of hypoxia in the tumor microenvironment when atovaquone inhibits OXPHOS and lowers oxygen consumption by cancer cells. These observations are well supported by data from a Phase I clinical trial (ATOM, NCT02628080) where atovaquone reduces tumor hypoxia in patients with non-small cell lung cancer [16, 44]. Our experiments provide an alternative, but potentially complementary, mechanism for PD-L1 upregulation by atovaquone. Our mechanistic studies for the first time link PD-L1 expression to oxidative damage and the subsequent activation of DDR by atovaquone. We demonstrate atovaquone-induced PD-L1 upregulation in vitro as well as in in vivo experiments. We further demonstrate that this upregulation of PD-L1 is directly triggered through the involvement of ATM/ATR and STAT-1 signaling. Our in vitro experiments did not implicate IRF-1, p53, and HIF-1α for the increase in PD-L1 in atovaquone treated cancer cells.

While the data from the CT26, 4T1 and the ID8-F3 models are promising, it must also be noted that additional benefit of atovaquone-anti-PD-L1 combination therapy was not observed in MC38 and LLC tumor models [43]. We propose that these seemingly discrepant results can be explained by considering the multiple physiologic outcomes triggered by atovaquone. DNA damage and ER stress caused by atovaquone contribute to a cellular catastrophe that results in cancer cell death [9, 21, 24, 45]. However, while these events are underway, the cancer cell is also in a “damage-control” mode- elevating the pentose phosphate pathway, thereby generating glutathione to neutralize the oxygen radical flux induced by atovaquone. Similarly, we now propose that the immune recognition afforded by DAMP expression is countered by hypoxia reduction [43] and DDR-mediated upregulation of PD-L1 to suppress T cell recognition. The outcome of atovaquone therapy is therefore determined by the balance between these varied physiologic reactions developing in the cancer cells. Those tumors with ability to express higher levels of PD-L1 as compared to DAMPs are likely to respond better to atovaquone plus anti-PD-L1/anti-PD-1 combination therapy. These mechanistic insights are key to designing future clinical trials where decisions to use atovaquone as a single agent or in combination with anti-PD-L1/anti-PD-1 or other immune therapies are based on the ability and extent to which the tumors engage the compensatory responses to OXPHOS inhibition by atovaquone.

## Supplementary Material

Supplementary Files

This is a list of supplementary files associated with this preprint. Click to download.

- supplemenatryfinalfigures02172026.pptx
- Table1phenotypiccharacteristicsofliverhistologystudiedfortoxicitystudiesinthecombinationtreatmentstudy..xlsx
- SupplementaryTable1AntibodyList.xlsx
- SupplementaryTable2ReagentList.xlsx
- SupplementaryTable3Nuclearparameters.xlsx
- Uneditedwesternblots.docx
